# Socio-demographic Factors Associated with Inhabitants’ View Related to Vaccines in Indonesia

**Published:** 2017-05

**Authors:** Harapan HARAPAN, Alma ALLETA, Samsul ANWAR, Abdul M. SETIAWAN

**Affiliations:** 1. Medical Research Unit, School of Medicine, Syiah Kuala University, Banda Aceh, Indonesia; 2. Tropical Disease Center, School of Medicine, Syiah Kuala University, Banda Aceh, Indonesia; 3. Dept. of Microbiology, School of Medicine, Syiah Kuala University, Banda Aceh, Indonesia; 4. Dept. of Family Medicine, School of Medicine, Syiah Kuala University, Banda Aceh, Indonesia; 5. Dept. of Statistics, Faculty of Mathematics and Natural Sciences, Syiah Kuala University, Banda Aceh, Indonesia; 6. Dept. of Microbiology, Faculty of Medicine and Health Sciences, State Islamic University Maulana Malik Ibrahim, Malang, Indonesia

## Dear Editor-in-Chief

Despite increasing vaccine coverage level, Indonesia is still in distance with target of Global Vaccine Action Plan (GVAP) (1). In 2012, Indonesia was the third place of countries with most unvaccinated infants with three doses of diphtheria-tetanus-pertussis (DTP3) (2) and the achievement on the National Immunization Program (NIP) was not evenly distributed across provinces-only eight provinces reached the national target of Universal Coverage Level (UCI) (1). Aceh province, the westernmost province of Indonesia, rank in the bottom six of UCI status in 2013 (1) and the lowest district in Aceh only gained 39% of UCI, far from national target of 100% (3). Therefore, understanding of factors that hinder or support the likeliness of vaccination among Acehnese inhabitants is an important sight for vaccination strategy in the future.

The aim of this study was to assess the socio-demographic factors influencing Acehnese inhabitants’ view related to vaccine. A cross-sectional survey was conducted during Nov 2014 to Mar 2015 and Aug to Dec 2015 in 11 regencies of Aceh. A set of validated questionnaires was used to assist the interviews. To elicit the view related to vaccine, two questions regarding the importance of the vaccines to prevent diseases and the safety of the vaccines for children were asked with responses on a Likert-type scale ranging from “1=strongly disagree” to “5=strongly agree”. The view related to vaccine was dichotomized into “good” and “poor” based on an 80% cut-off point. A logistic regression analysis was employed to analysis the data.

We included data of 1059 participants in the final analysis. Approximately, 33% of the participants had a poor view regarding vaccines. Education, occupation, monthly income, and economic status were associated with the participants’ perspective regarding vaccine while age, gender, marital status and type of residency had no association (Table 1).

**Table 1:** Socio-economic characteristics influencing participants’ view regarding vaccines (Good *vs.* Poor) (*n* = 1,059)

**Variable**	**n (%)**	**Good view (%)**	**Univariate logistic regression**	
			**OR (95% CI)**	***P*–value**
Age group (yr)				0.248
17–29 *(R)*	491 (46.4)	336 (68.4)	1	
30–44	395 (37.3)	272 (68.9)	1.02 (0.76–1.35)	0.891
45–59	150 (14.2)	91 (60.7)	0.71 (0.48–1.03)	0.078
60–84	23 (2.1)	14 (60.9)	0.72 (0.30–1.69)	0.449
Gender				
Male *(R)*	374 (35.3)	241 (64.4)	1	
Female	685 (64.7)	472 (68.9)	1.22 (0.93–1.59)	0.139
Education attainment				<0.001*
Primary school *(R)*	90 (8.5)	40 (44.4)	1	
Junior high school	106 (10.0)	48 (45.3)	1.03 (0.58–1.82)	0.906
Senior high school	414 (39.1)	284 (68.6)	2.73 (1.71–4.24)	<0.001**
Diploma	197 (18.6)	149 (75.6)	3.88 (2.28–6.57)	<0.001**
Graduated	252 (23.8)	192 (76.2)	4.00 (2.41–6.64)	<0.001**
Types of occupation				<0.001**
Farmer *(R)*	206 (19.5)	103 (50.0)	1	
Student/university student	171 (16.2)	100 (58.5)	1.408 (0.93–2.12)	0.101
Entrepreneur	210 (19.8)	140 (66.7)	2.00 (1.34–2.97)	0.001*
Civil servant	214 (20.2)	162 (75.7)	3.11 (2.05–4.71)	<0.001**
Private employee	171 (16.1)	138 (80.7)	4.18 (2.61–6.67)	<0.001**
Housewife	87 (8.2)	70 (80.5)	4.12 (2.26–7.47)	<0.001**
Marital status				0.143
Unmarried *(R)*	340 (32.1)	217 (63.8)	1	
Married	686 (64.8)	476 (69.4)	1.28 (0.97–1.69)	0.073
Widow	33 (3.1)	20 (713)	0.87 (0.41–1.81)	0.714
Monthly household income				<0.001**
<1 million IDR *(R)*	516 (48.7)	310 (60.1)	1	
1 – ≤ 2 million IDR	268 (25.3)	187 (69.8)	1.53 (1.12–2.10)	0.008*
2 – ≤ 3 million IDR	170 (16.1)	130 (76.5)	2.16 (1.45–3.20)	<0.001**
> 3 million IDR	105 (9.9)	86 (81.9)	3.01 (1.77–5.09)	<0.001**
Type of residency				
Suburb *(R)*	751 (70.9)	502 (66.8)	1	
City	308 (29.1)	211 (68.5)	1.08 (0.81–1.43)	0.600
Economic status				<0.001**
Poorest quintile *(R)*	210 (19.8)	118 (56.2)	1	
2^nd^	214 (20.2)	140 (65.4)	1.47 (0.99–2.18)	0.052*
3^rd^	214 (20.2)	145 (67.8)	1.64 (1.10–2.43)	0.014*
4^th^	209 (19.7)	149 (71.3)	1.94 (1.29–2.90)	0.001*
Richest quintile	212 (20.1)	161 (75.9)	2.46 (1.62–3.73)	<0.001**

CI: confidence interval, IDR: Indonesian rupiah, OR: odds ratio, R: reference group

* Significant at 0.05

** Significant at 0.001

Low education, working as farmer, low economic status and low monthly income were associated with a poor view related to vaccine. Therefore, these groups should be targeted to increase vaccination coverage in the future. One of the visible efforts is to increase the knowledge and attitudes regarding vaccination and this could be implemented using *Puskesmas* (community health center)-, hospital-, and *Masjid* (mosque)-based approaches (4).

In addition, one of the focus groups that should be targeted is that of student/ university students, as their views regarding vaccine have no difference compared to farmers. Therefore, intensive vaccination campaigns focusing on those groups might be required in the future to enhance the correct understanding of vaccination.

## References

[1] Ministry of Health Republic of Indonesia. Pokok-pokok hasil RISKESDAS Indonesia tahun 2013. Department of Research and Development Ministry of Health Jakarta, 2013.

[2] UNICEF Global and rregional iimmunization coverage (1980 – 2012). 2013 Available from: http://www.unicef.org/immunization/files/Global_immunization_coverage.pdf

[3] Aceh Provincial Health Office Profile kesehatan Aceh 2014. Banda Aceh, 2014.

[4] Harapan H Anwar S Setiawan A Sasmono R Aceh Dengue Study (2016). Dengue vaccine acceptance and associated factors in Indonesia: A community-based cross-sectional survey in Aceh. Vaccine, 34:3670–3675. 27208588 10.1016/j.vaccine.2016.05.026

